# Cretins and Cretinism

**Published:** 1857-01

**Authors:** 


Art. III.—*Cretins and Cretinism:* A Prize Thesis of the University of Edinburgh. By George S. Blackie, M.D., Curator of the Botanical Society of Edinburgh. Edinburgh, 1855: 8vo. pp. 70.

It is generally known, at the present day, that the great degeneracy of the human race, designated by the name of Cretinism, from the Latin word *Creatura*, is spread over the greater part of the valleys of the Alpine chain, which traverses several of the European countries. The honor, however, of ameliorating the sad condition of this unfortunate class of persons, was reserved for Dr. Guggenbühl, who (as we are informed by Dr. George S. Blackie, of Edinburgh, in his valuable and excellent treatise on Cretinism) "first formed the idea of establishing an abode for cretins, and place for their relief and cure." Let us throw a cursory glance over the life of this great philanthropist, to enable us to understand the laudable and praiseworthy motives, which induced him to devote his entire energies to the accomplishment of that great aim—"the restoration of the cretins to the form and condition of men."

Dr. Guggenbühl was born at Milan, in Canton Zurich, in 1816. From his earliest childhood, he evinced a decided inclination for medicine; and at the age of sixteen years, occupied himself in gathering plants, for the purpose of obtaining information of their medicinal properties. At the gymnasium of Zurich, he studied medicine and the natural sciences, under Schœnlein and Ocken, and philosophy under Troxler. Wishing to learn the primitive manners of the people, the natural products, and the diseases incident to the country, Dr. Guggenbühl, in 1836, travelled among the High Alps. At Zeedorff, in the Canton Uri, he observed an unfortunate cretin, muttering a prayer before a cross. He followed him into his cabin, when the mother told him her son's pitiable condition. This sight first gave an impetus to the philanthropic motive, which had heretofore lain dormant in the heart of this noble man; and from that moment, he determined that, by the grace of God, he would devote his life and energies to the great cause he had in view, saying, that "it was better to save one human soul, than to acquire all earthly treasures." Having finished his medical studies, he resided for two years at Cleinthal, in the Canton Glaurus, where his whole time was devoted to the study of cretinism. By a second tour through the mountainous parts of Switzerland, he was able to make an estimate of their immense number. It was at this time that he resolved to mature his plan of forming an establishment for the treatment of cretins. Many obstacles were thrown in his path. He was obliged to contend with envy and ignorance. In Canton Berne, they even went so far as to say that these unfortunate beings were indeed only animals, and that it was greatly to be regretted that a young man should consume his strength in so fruitless an object. Not allowing himself to be disheartened by these attacks, Dr. Guggenbühl placed the subject before the Swiss Association for the Advancement of Science, and, after much perseverance, and with the invaluable assistance of Emanuel de Fellenberg, of Hofwyl, and that of Dr. Mederer, of Geneva, a pupil of Pestalozzi, his desires were finally accomplished. His next object was to seek a desirable locality for his hospital; and, knowing that the greatest advantages were to be derived from purity of atmosphere, he determined that it should be at as "high an elevation as practicable." He therefore chose the hill called Abendberg, near Interlachen, in Canton Berne; it is three thousand feet above the sea; and at this height, cretinism has never been known to occur. A more desirable locality could not possibly have been selected. Abendberg is in the midst of the chain of the High Alps, embosomed in the most beautiful scenery, which, even upon the minds of the most miserable, must leave a powerful and lasting impression. The view from this mountain, is one of the most exquisite in Switzerland. At its base lie the two unruffled lakes of Thon and Brienz, in whose clear surfaces the highest ridges of Oberland are mirrored. In the distance rises the queen of mountains, the beautiful Jungfrau, well guarded by her two gloomy companions, the Eiger and the Monk. Nature is pre-eminently glorious in this unrivalled region: this is acknowledged to be true by all who have seen it; and even the "blunted minds of the young cretins, as they open to a consciousness of their existence," seem to recognize its great beauty. Such was the spot upon which was erected the first cretin hospital, an institution designed for the amelioration of this most unfortunate class of beings; and how grateful should all humanity be to that man, whose untiring efforts and almost incredible perseverance finally won for him the desired accomplishment of his benevolent enterprise! In describing this hospital, Dr. Blackie says, "that it consists of about twenty rooms, among which are three large halls, a bath-room, and hall for gymnastic exercises in the winter-season. All the rooms can be heated, and the dormitories are supplied with ventilators. The kitchen has an iron kitchen-range, and a basin for supplying the baths. Two fountains supply them with a pure, excellent drinking-water, which can be collected in two reservoirs." At a later period a second building was added, intended as a residence for the mothers who wished to learn the necessary treatment of their deformed children. In 1842, an English philanthropist of London visited Abendberg, and spoke enthusiastically of this new establishment and its great success, in a pamphlet, entitled, "Some Account of Cretinism, and the Institution for its Cure, on the Abendberg, near Interlachen, in Switzerland." Dr. Twining, the gentleman alluded to, used his utmost exertions to engage all good-hearted and charitable Englishmen to sustain this noble work, as Switzerland, which numbers several thousand cretins, was not able to furnish the necessary funds. Dr. Twining will ever be honored by posterity for the zeal which he evinced in this cause; his latter years were consecrated to it, and in his dying hour his thoughts still wandered to Abendberg, which, even in such a moment, maintained its strong hold upon his affections. His father, Richard Twining, also took an active part in the civilization of the cretins; and nearly all that has been done of late in England toward the improvement of the condition of idiots is justly due to the repeated and persevering exertions of these philanthropists.

Cretinism is perhaps more prevalent in the Alps of Switzerland than in any other portion of Europe. It was first noticed by Platner, about the middle of the seventeenth century, as occurring among the peasants in Carinthia and the Valois. It was afterwards found in a greater degree in other valleys of Switzerland.

M. de Saussure, in his "Voyages en Alpés," observes that it occurs most frequently in the Valley of Aosta, at the foot of Mont Blanc. A valley of Canton Argovie contains twenty-eight villages infected with cretinism. At the commencement of this century there was not one cretin in the village of Buchs, while at the present day there are upwards of fifty. At Martigny the disease is visibly decreasing; at Viége it has already diminished one-third, the number of its cretins being now about thirty; there are perhaps almost as many in the neighboring village of Mundt, which consists of 200 inhabitants. According to Dr. Blackie, "among the 2,188,000 souls who form the population of the Swiss Confederation, no less than 20,000 persons suffer in a greater or less degree from this disease."

Nor are France and Norway exempt from it. In various parts of Rhenish Prussia and in Austria, it is very common. In Upper Austria, it is so prevalent that "entire families consist alone of cretins;" and upon examination 6000 were found in Steiermark. The disease is also endemic in the highlands of Bavaria and in Denmark. The number of cretins in the kingdom of Sardinia is supposed to be upwards of 10,000. In Algeria, and in China, among the Himalayan Mountains, they are frequently found. But it is of most frequent occurrence in the valleys of Switzerland, and it is there, as we have already seen, that active measures have been taken for the promotion of a durable and well-established cure.

Let us now glance at the various hypotheses regarding the origin of this disease. By the word cretin we understand "a being deformed and distorted, an abortion of man, peculiarized by a pale, leaden color of the face, by a flaccidity of the flesh, an unexcitable nature, an extraordinary amount of laziness and inactivity, and inability to speak or utter articulate sounds, and generally with very large goitres, a circumstance which has led to a much misunderstood connection of the two complaints." Dr. Guggenbühl defines cretinism to be "a disease of the cerebro-spinal system, causing a want of development of the body and perceptive faculties, and considers that if this disease of body be removed, the maladies of the mind will be more easily overcome than in those cases in which the body is in full vigor and the mind idiotic." Dr. Guggenbühl divides cretins into four classes: 1. The atrophied cretins; 2. The rickety cretins; 3. The hydrocephalic cretins; 4. The congenital cretins. Dr. Blackie tells us that by some the cause of cretinism is attributed "to a cachectic state of the body, which, inducing scrofulous rickets of the cranial bones, and causing them to press on their nerves at their origin, produced the phenomena of idiotcy;" an opinion in which Dr. Blackie seems fully to concur.

With some, cretinism first shows itself in a stunted growth of body; with others, the body is well formed, but the mind totally extinguished; some are able to hear, but cannot speak; and it very often occurs, that cretins are both deaf and dumb, and that their body and mind are equally degenerate. The appearance of this unfortunate class of persons is, at times, most revolting, and travellers passing through the valleys of Switzerland, have often been unable to distinguish them from the brutes.

The first symptoms of cretinism is regarded by Kohl to be a dulness of the eye, by which one is able to tell whether or not the child is idiotic. When this makes its appearance, the countenance becomes inanimate, the legs bend inward, and it is only with great difficulty and awkwardness that the child can walk. Thought and feeling gradually fade away, until finally he becomes a perfect cretin. His entire being is changed. All human instinct seems to have disappeared, and he becomes malicious and cruel. We will quote a part of the description of the cretins given by M. Fodéré, in his "Traité du Goître et du Crétinisme:" "The smallness of their heads," he writes, "is disproportioned to the rest of the body; their heads are flattened at the summit and at the temples, and the tuberosity of the occiput is less projecting than natural; their eyes are small, sometimes deeply sunk, at others prominent; their look fixed and stupid; their chests are flat; their fingers thin and long, with small articulations; the soles of their feet flattened, and sometimes bent. When he has attained his full stature, which is from thirteen to sixteen decimetres, the cretin's skin becomes brown, his insensibility continues obtuse, he is indifferent to cold or heat, or even to blows and wounds, and is generally deaf and dumb. Cretins often eat with avidity raw onions, and even charcoal, which proves that the organ of taste is gross and imperfect." There has been great diversity of opinion regarding the longevity of cretins. It is asserted by Kohl, that their miserable lives are often prolonged to upwards of seventy years; the generality of persons, however, believe that it is not uncommon for them to live thirty years, and that, upon attaining this age, they generally die; and most truly do we hope this to be the case, for the approach of death must illumine their darkened, chaotic minds, with a ray of pleasure, the "one pearl," it may be, of their inanimate existence, bringing with it an intense longing for the severing of the leaden chain which binds them to this earth, where they have lived,

"Deformed, crooked, sere,
Ill-faced, worse-bodied, shapeless everywhere."

We may now investigate the causes of cretinism. Some have attributed its origin to goitre; but this opinion has been proved groundless, as, in many regions where almost every person is goitrous, cretins have been found to be entirely free from it. Others again have ascribed it to the use of snow water, and water impregnated with calcareous earth. These opinions are also without foundation. We know that persons born near the glaciers drink no other water than that obtained by the melting of ice and snow, and they are rarely, if ever, subject to the disease. Again, the common water of Switzerland is not impregnated with calcareous substances, but surpasses almost all other water in purity and flavor. By others it has been assigned to an impure and humid atmosphere. This belief was originated by M. de Saussure. He affirmed that water was by no means one of the causes of the disease, but that it ought to be attributed almost entirely to the heated and corrupted air, which the inhabitants of the valleys are compelled to breathe. Dr. Blackie, in his "Treatise on Cretinism," says that Dr. Morel, physician to the Hospital of Mauville, considers cretinism to be caused by the reception of a deleterious miasma into the constitution; and also believes that the absence of iodine in the water may be conducive to it, as in his analysis of the waters in cretinous countries, he found carbonate of lime, chloride of lime, silica, potash, nitrate of ammonia, sulphate of lime, magnesia, and other matters, but never iodine. This disease arises from a multiplicity of causes, and cannot, therefore, be attributed to any single one. It is never found in the higher villages, those situated about three or four hundred feet above the level of the sea. It is only upon descending that cretins are found, and upon reaching the plains they disappear altogether. Many of the streams which traverse the minor valleys flow into the Rhone, and in their downward course often leave behind matters which distribute a pestilential air through the country; this, together with the fact that in these villages cretins are exposed for four months of the year to the rays of a scorching sun, and of their being an excessively indolent and immoral people, has given rise to the supposition of M. de Rambuteau. He observes, moreover, that the disease is not hereditary; for strangers coming to reside in an infected locality, are as subject to it as are the indigenous inhabitants.

Let us, finally, take a slight survey of the mode of treatment used by Dr. Guggenbühl on the Abendberg. Dr. Twining observed that it consists in improving the bodily health by air, exercise, diet, frictions, baths, and medicine, and subsequently rousing the inert senses by a steady course of instruction. The bodily health is first attended to; and by the aid of the fresh mountain air, cold baths, and proper food, many children, who, upon their entrance into the institution, could not hold up their heads or move their limbs, have been enabled to walk steadily and exert their muscles. When the health is sufficiently improved, education is attended to. Ear-trumpets are used to awaken the sense of hearing. The child is then taught to perform with its mouth, the motions required to express the sound impressed upon the mind. Letters carved in wood are then placed before them, and from these they gradually learn to form words. When other methods fail, letters are figured in phosphorus on the walls of a darkened room, and this plan has often proved effectual in impressing the letters indelibly upon their awakening minds. They are then taught to distinguish forms, colors, &c. Cretins are generally fond of music; and "hearing is exercised by means of bells, singing, and instrumental music." They afterwards learn to read and write; arithmetic being acquired by means of the counting-frame. After this has been attained, the girls are taught to sew, and the boys to garden.

Dr. Guggenbühl has conducted his establishment for fifteen years with the most happy results, and so well has his mode of treatment succeeded that one-third of the cretins who have resided in the hospital have been completely restored to mental and bodily activity.

The benevolent example set by Dr. Guggenbühl has stimulated many other noble-hearted men with the desire of founding similar institutions for the amelioration of cretins and idiots. A number have already been established in Austria, Prussia, Wurtemberg, Saxony, France, Bavaria, Baden, and Sardinia; and in England and Scotland there are several hospitals for idiots.

Fortunately this dreadful malady does not exist in the United States, except in the most limited degree, and in localities corresponding in climatic condition with the cretinous regions of the Old World. In New England many idiots are found; and at Barre, Massachusetts, is an admirable institution for their treatment, under the superintendence of the distinguished philanthropist, Dr. Howe, formerly of Boston.

In taking leave of this subject, we return our cordial acknowledgments to Dr. Blackie for the pleasure we have derived from the perusal of his excellent monograph. It is alike creditable to his talents, and to the University of which he is a graduate. We hope that it is only a prelude to other and more elaborate emanations from his able and graceful pen.

